# Risk factors for CBCT-defined condylar cortical blurring and regional osseous changes in temporomandibular disorders

**DOI:** 10.1097/MD.0000000000049733

**Published:** 2026-07-17

**Authors:** Xinbang Huang, Yu Wang, Yong Li, Hailei He, Biaodong Li, Lingfan Zhao

**Affiliations:** a Department of Oral and Maxillofacial Surgery, Ganzhou People’s Hospital, Ganzhou, Jiangxi Province, China.

**Keywords:** bone remodeling, cone-beam computed tomography, degenerative joint disease, mandibular condyle, parafunctional oral behaviors, risk factors, Temporomandibular disorders

## Abstract

Temporomandibular disorders (TMD) are commonly accompanied by adaptive or degenerative osseous alterations of the mandibular condyle, and parafunctional oral behaviors may contribute to abnormal joint loading. This retrospective cross-sectional study investigated the association between self-reported parafunctional activities and cone-beam computed tomography (CBCT)–defined condylar remodeling patterns in adults with TMD. Consecutive patients diagnosed according to the Diagnostic Criteria for Temporomandibular Disorders (DC/TMD) between June 2023 and June 2024 were reviewed, and 102 individuals with complete clinical and imaging data were included. Parafunctional behaviors were assessed using a structured questionnaire adapted from the Oral Behaviors Checklist, focusing on unilateral chewing, tooth clenching, bruxism, and preference for hard foods. Bilateral CBCT images were reconstructed in multiplanar views, and osseous changes (including cortical blurring/vanishing, defects, flattening, sclerosis, osteophytes, and cystic degeneration) were recorded and localized by quadrant. Multivariable logistic regression was used to identify behavioral factors independently associated with specific radiographic phenotypes. Hard-food preference was independently associated with cortical blurring/vanishing and with changes involving the anterolateral and posterolateral condyle as well as the articular tubercle. Habitual unilateral chewing showed an inverse association with anteromedial condylar changes. Bruxism and clenching did not remain significant after adjustment. These findings suggest that specific parafunctional behaviors, particularly hard-food preference, are associated with distinct CBCT-detected condylar remodeling patterns in TMD and support behavioral assessment and counseling during clinical evaluation.

## 1. Introduction

Temporomandibular disorders (TMD) refer to a group of musculoskeletal and neuromuscular disorders affecting the temporomandibular joint (TMJ), the masticatory muscles, as well as soft and hard tissues, that are associated with pain, restricted function, and/or audible sounds from the joint.^[1]^ The TMD is the only fully movable joint in the craniofacial complex and consists of the temporal articular surface, the mandibular condyle, an articular disc, and ligaments and capsule.^[2]^ The thick biconcave disc is connective tissue that is primarily dense fibrous connective tissue, varies in thickness, and connects anteriorly with the lateral pterygoid muscle.^[3]^ The Diagnostic Criteria for Temporomandibular Disorders (DC/TMD) developed by Schiffman et al in 2014 describes TMD as pain-related or intra-articular types.^[4]^ A number of studies have identified an association between TMD-related pain and various structural features observed by magnetic resonance imaging (MRI), including disc displacement, condylar degeneration, joint effusion, and deformation.^[5–7]^ Unfortunately, little of the aforementioned research has examined imaging features and functional signs and symptoms, such as joint clicking and limited opening, which are also part of the TMD symptom complex.

The condyle has an important role in the development of the mandible and in mandibular function; it bears the functional load associated with mastication and contributes to the morphology and stability of the TMD. Structurally, the condyle consists of cartilage and subchondral bone that interact synergistically to dissipate and distribute applied mechanical loads during mandibular movements.^[8]^ Condylar morphology and subchondral bone structure are influenced by age and mechanical loading stress. Age results in structural adaptations characterized by a thinning cortex and lower trabecular densities within existing subchondral bone. Adaptive remodeling likely occurs in response to long-term mechanical loading stress.^[9,10]^ Adaptive remodeling produces changes in condylar morphology contributing to the diversity of condylar shape, and this differs between individuals and between sides (right/left) on the same individual. Excessive functional loading or overload from habitual parafunctional activities can result in pathological remodeling, which can result in condylar resorption and/or degenerative changes consistent with osteoarthritis.^[11]^

Several studies have examined the associations between parafunctional oral behaviors and TMD symptoms; however, few studies have examined the imaging manifestations of parafunctional oral behaviors, which exist in the same patients as the reported symptoms, or their manifestations using cone-beam computed tomography (CBCT). CBCT is three-dimensional imaging and provides high spatial resolution capabilities of osseous structures, which adds detail to how condylar morphology can be characterized and to the possible early recognition of remodeling.^[12,13]^ Oral behavioral activities are defined as all mandibular activities/behavior that occur outside of normal chewing, swallowing, or speaking functions. These may include bruxism, biting down or clenching, abnormal sucking, and habitual unilateral chewing. When conducted, these behaviors should be assessed and documented utilizing the Oral Behaviors Checklist (OBC-21), to provide a total score regarding how the behaviors affect the loading and stress on the TMJ.^[14]^ These activities change intra-articular pressure and create local mechanical strain within the TMD. This may ultimately lead to pathological remodeling or degenerative changes.^[15,16]^

Significant recent gains have been recognized and understood as perhaps behavioral contributions to TMD pathology; however, whether parafunctional oral behaviors are associated with region-specific radiographic manifestations of condylar remodeling on CBCT remains insufficiently investigated.

Therefore, this study aimed to examine the association between parafunctional oral behaviors and the type and anatomical distribution of CBCT-detected condylar bone changes in patients with TMD. We hypothesized that specific oral behaviors, particularly hard-food preference, bruxism, clenching, and habitual unilateral chewing, would be associated with distinct regional patterns of condylar bone remodeling.

## 2. Information and methods

### 2.1. Study design and population

This study was approved by the Ethics Committee of Ganzhou People’s HospitalThis retrospective cross-sectional study included consecutive adult patients with TMD who visited the Outpatient Department of Oral and Maxillofacial Surgery, Ganzhou People’s Hospital, between June 2023 and June 2024. This study conducted on 102 patients diagnosed with Temporomandibular Disorders (TMD) who were admitted to our institution between June 2023 and June 2024. All participants were diagnosed according to the *Diagnostic Criteria for Temporomandibular Disorders (DC/TMD*).^[1]^ The sample size was estimated for logistic regression analysis using an α level of 0.05 and 80% statistical power to detect an odds ratio (OR) of at least 2.0 for the association between parafunctional oral behaviors and CBCT-detected condylar bone changes. Based on preliminary clinical records and previous behavioral studies in TMD populations, the expected exposure proportion of major oral behaviors was assumed to be approximately 20 to 30%. Considering the retrospective availability of complete clinical, questionnaire, and CBCT data, 102 eligible patients were finally included. This study was approved by the Institutional Medical Ethics Committee of Ganzhou People’s Hospital. Because this was a retrospective review of existing clinical, questionnaire, and imaging records, the requirement for written informed consent was waived by the ethics committee. All patient data were anonymized before analysis.

#### 2.1.1. Inclusion criteria

1. Age ≥ 18 years.
2. Diagnosis of TMD according to DC/TMD clinical criteria.
3. Availability of CBCT examination of bilateral TMDs.
4. Complete clinical data and questionnaire records.

#### 2.1.2. Exclusion criteria

1. Neurological, psychiatric, or intellectual developmental disorders.
2. Severe hepatic, renal, or cardiac insufficiency.
3. Pregnancy or lactation.
4. Autoimmune or connective tissue disease.
5. Severe infection or critical illness.
6. Congenital craniofacial deformity or history of trauma.
7. Systemic diseases such as rheumatoid arthritis.

### 2.2. Diagnostic criteria and examiner reliability

All patients were evaluated by trained clinicians following DC/TMD Axis I clinical protocols. A total of 50 consecutive patients were first assessed to establish examiner reliability, and inter-examiner agreement achieved a Kappa value of 0.728, indicating good diagnostic consistency.

### 2.3. Questionnaire on oral behaviors

Oral behaviors were assessed using a structured questionnaire adapted from the Oral Behaviors Checklist-21 item version (OBC-21). The OBC-21 is commonly used to record the frequency of awake and sleep-related oral behaviors, including clenching, grinding, unilateral chewing, and other nonfunctional mandibular activities. Each item was scored on a 5-point frequency scale from 0 to 4, where higher scores indicated more frequent oral behavioral activity and potentially greater functional loading of the TMJ. In the present study, the questionnaire showed acceptable internal consistency, with a Cronbach α value of 0.81.

### 2.4. CBCT imaging protocol

Bilateral TMD imaging was performed using a dental CBCT scanner (MCT-1, Ex-2F, Morita, Japan) at 90 kVp (kilovolt peak), 10 mA (milliampere), voxel size 0.2 mm. During scanning, participants maintained maximum intercuspation without movement. The acquired data were reconstructed using i-Dixel software, producing multiplanar views (sagittal, coronal, and axial).

#### 2.4.1. Image analysis

The condyle and articular fossa were divided into 4 quadrants (anterolateral, anteromedial, posterolateral, and posteromedial). Radiographic changes were classified as blurred/vanishing cortex, defect/destruction, condylar flattening, osteosclerosis, osteophyte formation, or cystic degeneration, based on TMD osteoarthritic changes reported in previous literature.^[2]^ Two independent radiologists, blinded to clinical data, evaluated all CBCT scans. A finding was considered positive only when observed in at least 2 consecutive layers. Inter-observer agreement was excellent (Kappa = 0.709; intraclass correlation coefficient = 0.872). A repeat reading after one month yielded a repeatability Kappa value of 0.864.

### 2.5. Statistical analysis

Data were analyzed using Statistical Package for the Social Sciences version 21.0 (IBM Corp., Armonk). The distribution of continuous variables was assessed using the Shapiro–Wilk test and visual inspection of histograms. Normally distributed continuous variables were expressed as mean ± standard deviation and compared using the independent-samples *t*-test. Non-normally distributed variables were expressed as median and interquartile range and compared using the Mann–Whitney *U* test. Categorical variables were presented as frequencies and percentages and compared using the chi-square test or Fisher exact test, as appropriate. Continuous variables were expressed as mean ± standard deviation, while categorical data were presented as frequencies and percentages. Univariate analysis was performed using the independent-samples t-test for continuous data and the chi-square test for categorical data. Variables with *P* < .05 in univariate analysis were entered into a multivariate binary logistic regression model to identify independent risk factors. ORs and 95% confidence intervals (CIs) were calculated. Statistical significance was set at *P* < .05.

## 3. Results

### 3.1. Demographic characteristics

The study sample of 102 individuals total (54 males and 48 females) was selected for this retrospective cross-sectional study. The mean age was 38.5 ± 16.2 years, and the mean symptom duration was 12.8 ± 27.9 months (Table 1). All the individuals met the DC/TMD diagnostic criteria.

**Table 1:** Basic characteristics.

Variable	Value
Total patients	102
Age, mean ± SD (yr)	38.5 ± 16.2
Gender (male/female)	54/ 48
Disease duration (mo), mean ± SD	12.8 ± 27.9

SD = standard deviation.

### 3.2. Distribution of parafunctional oral behaviors

Of the 102 clinical cases assessed, 22 patients (21.6%) exhibited bruxism (clenching and/or grinding), 23 patients (22.5%) reported clenching only, 70 (68.6%) reported unilateral chewing, and 18 (17.6%) preferred hard food (Table 2). Several patients displayed more than one parafunctional behavior simultaneously. Condylar cortical bone remodeling was most likely to be seen in patients with unilateral chewing (74.3%) and hard food preferences (77.8%); bruxism alongside condylar changes occurred 72.7% of the time, and clenching only occurred 65.2% of the time.

**Table 2:** Type of bone change with TMJ (case/%).

Bad habit	Number of patients with bone changes [cases (%)]			
	n (case)	One-sided	Two-sided	Total
Bruxism	22	6 (27.27)	10 (45.45)	16 (72.72)
Clench teeth	23	6 (26.09)	9 (39.13)	15 (65.22)
Unilateral chew	70	24 (34.29)	28 (40.00)	52 (74.29)
Likes to eat hard things	18	1 (5.56)	13 (72.22)	14 (77.78)

TMJ = temporomandibular joint.

### 3.3. Imaging findings and types of condylar bone changes

CBCT abnormalities indicative of condylar bone remodeling occurred in the form of blurred or vanishing cortical bone, defect/destroyed, flattened, osteosclerotic, osteophyte, and cystic changes (Table 3). The most common condylar change reported was flattening in 24.3% of joints, and flattening and sclerotic changes were most commonly seen in patients with unilateral chewing or hard food preferences; this suggests that repeated mechanical loading may predispose to adaptive or compensatory remodeling.

**Table 3:** Harmful oral habits and bone changes in the number of joints (case/%).

Bad habit	Bruxism (n = 44)	Clench teeth (n = 46)	Unilateral chew (n = 140)	Likes to eat hard things (n = 36)
Number of joints with bone change [%]				
Blurred and vanishing type	14 (31.82)	13 (28.26)	30 (21.43)	15 (41.67)
Defective and destructive type	6 (13.64)	4 (8.70)	21 (15.00)	7 (19.44)
Condylar flattening type	10 (22.73)	8 (17.39)	34 (24.29)	9 (25.00)
Osteosclerosis type	8 (18.18)	8 (17.39)	19 (13.57)	21 (14.58)
Osteosis type	6 (13.64)	10 (21.74)	21 (15.00)	8 (22.22)
Cystic type	1 (2.27)	2 (4.35)	6 (4.29)	0 (0)

### 3.4. Anatomical distribution of bone changes

Bone changes were most reported in the anterolateral condyle (42.9%) and articular tubercle (22.2%), while minimal changes occurred in the posterior and medial fossae (Table 4). The most common changes reported in the anterolateral condyle were associated with patients with habits of frequently eating hard food (55.6%), and bruxism and clenching were reported more evenly in the anterior-posterior locations.

**Table 4:** Harmful oral habits and bone changes in the condyle and glenoid fossa.

Bad habit	Bruxism (n = 44)	Clench teeth (n = 46)	Unilateral chew (n = 140)	Likes to eat hard things (n = 36)
Condyle				
Antero-external	14 (31.82)	16 (34.78)	60 (42.86)	20 (55.56)
Antero-	12 (27.27)	13 (28.26)	43 (30.71)	11 (30.56)
Postero-exterior	8 (18.18)	7 (15.22)	21 (15.00)	10 (27.78)
Posteroy	9 (20.45)	10 (21.74)	26 (18.57)	1 (27.78)
Articular fossa				
Antero-external	4 (9.09)	3 (6.52)	10 (7.14)	4 (11.11)
Antero-	7 (15.91)	2 (4.35)	9 (6.43)	2 (5.56)
Postero-exterior	0 (0)	1 (2.17)	2 (1.43)	0 (0)
Posteroy	0 (0)	0 (0)	0 (0)	0 (0)
Articular tubercle	5 (11.36)	4 (8.70)	13 (9.29)	8 (22.22)

### 3.5. Correlation and regression analysis

Pearson correlation analysis indicated a statistically significant correlation between hard-food preference and a blurred/vanishing cortical condylar bone (*R* = 0.31, *P* = .019). Following that, multivariate logistic regression was assessed to establish independent risk factors (Table 5). The observations from the study can be viewed as this: Hard food preference was positively associated with cortical blurring (OR = 2.57, *P* = .019) and also with bone changes in the anterolateral condyle (OR = 2.30, *P* = .025), posterior-lateral condyle (OR = 2.69, *P* = .023), and articular tubercle (OR = 3.07, *P* = .020). However, unilateral chewing was accompanied by a negative association with anterior-medial condyle changes (OR = 0.31, *P* = .013). Habitual unilateral chewing showed a negative association with anteromedial condylar changes. However, because of the cross-sectional design, this finding should not be interpreted as evidence of a protective or adaptive effect. It may reflect differences in loading direction, chewing-side preference, or unmeasured confounding factors, and should be verified in future longitudinal biomechanical studies. Bruxism and clenching were trending but did not reach significance (*P* > .05).

**Table 5:** The correlation between harmful oral habits and bone changes.

Index	Unilateral chew			Likes to eat hard things		
	OR	*P*	95% CI	OR	*P*	95% CI
Blurred and vanishing type	2.57	.019	1.167–5.658	–	–	–
Condyle-antero-external	–	–	–	2.298	.025	1.111–4.753
Condyle-postero-exterior	–	–	–	2.692	.023	1.145–6.332
Articular fossa-antero	–	–	–	0.311	.013	0.124–0.780
Articular tubercle	–	–	–	3.067	.02	1.189–7.908

CI = confidence interval, OR = odds ratios.

### 3.6. Summary of key findings

To summarize, the condylar remodeling patterns observed on CBCT suggested a significant association with parafunctional oral behaviors. The strongest independent risk factor was hard food preferences for blurring and anterior-lateral changes, while unilateral chewing had a negative association with anterior-lateral changes. Overall, there were no significant associations with sex or age.

## 4. Discussion

This study aimed to examine the relationships between oral behaviors and condylar remodeling identified through CBCT in individuals with TMJ disorders. Overall, the findings showed that hard-food preference was an independent risk factor for evidence of blurring or vanishing condylar cortex and changes in anterolateral bone, while unilateral chewing was negatively associated with changes in the anteromedial condyle, suggesting an adaptive biomechanical response instead of pathological remodeling.

### 4.1. Comparison to previous studies

TMD is a multifactorial condition that encompasses biological, psychosocial, and behavioral features. As previously indicated, the study findings suggest that parafunctional oral behaviors, including clenching and chewing hard foods, are significantly related to excessive mechanical loading and condylar remodeling.^[13–16]^ Mortazavi et al^[17]^ reported a significant relationship between bruxism and osseous changes related to TMD, and Ohrbach and Dworkin^[18]^ determined that repetitive joint stress increases degenerative processes of the TMD. Oluwajana et al^[19]^ and Palmer and Durham^[20]^ also determined that behavioral and psychosocial variables influence degenerative processes associated with TMD.

Furthermore, epidemiological data continues to support the findings between soft-food preference and increased prevalence of degenerative TMD. Pantoja et al^[21]^ concluded that “parafunctional or atypical oral habits and behaviors were identified in a high percentage of indicators with increasing degenerative TMD disease”; Perrotta et al^[22]^ likewise reported a high prevalence of parafunctional behaviors associated with extensive degenerative TMD. Aligned with these studies, we found that a hard-food preference significantly increased the likelihood that TMD condylar cortical changes would be present, while unilateral chewing was associated with condylar flattening – results^[23–26]^ comparable to those found by Rongo et al^[27]^ and Takahara et al.^[28]^

### 4.2. Imaging-based interpretation

CBCT has been established as a suitable imaging modality to assess osseous changes in TMD given its high resolution, 3D visualization, and low radiation dose.^[29–33]^ Our findings confirm that CBCT provides superior accuracy in identifying early remodeling attributes, such as cortical thinning, flattening, and sclerosis. Recent studies that have compared CBCT with MRI^[34,35]^ suggest that osseous and soft-tissue changes coexist, further suggesting that discovering early point-specific CBCT changes can be used as an early structural biomarker of joint overload. Compared to the above evidence, this study clarifies the association between CBCT-identified bony changes and self-reported ortho behavior, thus incorporating an anatomical-behavioral approach.

### 4.3. Clinical implications

This study has clinical implications worth noting. The assessment and modification of behaviors should be seen as essential to managing TMD. Recognizing patients who frequently clench or grind or have a habitual preference for hard food allows clinicians to intervene with early behavioral treatment, patient education, or dietary counseling. These behavioral management techniques are aimed at reducing TMD overload, the potential for change of bony remodeling over time, and potentially improving patient outcomes. Behaviors that are changed or reported as less impactful may promote the planning of future targeted individualized patient education or interventions for TMD overload.

### 4.4. Strengths of the study

A significant strength of the study was the use of standardized DC/TMD diagnostic criteria and OBC-21 behavioral assessment, both of which supported consistency in method and extrapolation of data. A secondary benefit of the study was using high-resolution CBCT images attesting to the detailed description of condylar morphology, allowing for identification of early changes within the bone that occurred potentially weeks or months before they were even apparent on the conventional radiograph.^[36,37]^ The association between reported oral behaviors and local bone remodeling characteristics are of translational importance clinically in order to identify patterns with distribution of stress locally.

### 4.5. Limitations and future directions

Although a valuable contribution to the clinical implications of TMD, the study has limitations. The study was conducted at a single site with a small sample size, which limits generalizability. It excluded most variables that contributed to TMD pathology, such as disc displacement, occlusal interferences, and malocclusion. The cross-sectional study design also limits any inferences of causality between behavioral habits and bone changes. Future studies to further determine the temporal and mechanistic relationships that exist and combine measures of MRI and finite-element biomechanical models are reported as beneficial to the education of oral behaviors.^[38,39]^ Because all participants were recruited from a single outpatient center, selection bias cannot be fully excluded, and the findings may not be generalizable to community-based or inpatient populations.

In summary, parafunctional oral behaviors, particularly preference for hard food and unilateral chewing, are significantly associated with differing CBCT-detected condylar remodeling patterns in patients with TMD. Emphasizing patient clinical assessments using behavioral assessment and CBCT may assist in understanding early patterns of TMD overload and preventive care. Behavioral management and patient education, targeted to prevention, may have a significant impact on decreased functional stress and mitigation of degenerative TMD changes. The negative association between unilateral chewing and anteromedial condylar changes should be interpreted cautiously. Although it may reflect region-specific differences in mechanical loading, the present cross-sectional data cannot establish whether this represents adaptation, protection, or confounding.

## 5. Conclusion

Certain parafunctional oral behaviors, namely chewing on one side only and preference for harder foods, were related to specific patterns of condylar bone remodeling, as assessed by CBCT. The findings offer a preliminary set of target behaviors around which to counsel on prevention and early clinical intervention activities in individuals with temporomandibular disorders (TMD). Given the cross-sectional design and limited sample size, however, additional studies, prospective and multicenter, are necessary to establish the associations, understand causations, and gain greater clarity about the biomechanical and biological pathways relating behaviors to condylar remodeling.

## Author contributions

**Conceptualization:** Xinbang Huang, Yu Wang, Yong Li, Hailei He, Biaodong Li, Lingfan Zhao.

**Data curation:** Xinbang Huang, Yu Wang, Yong Li, Hailei He, Biaodong Li, Lingfan Zhao.

**Formal analysis:** Xinbang Huang, Yu Wang, Yong Li, Hailei He, Biaodong Li, Lingfan Zhao.

**Funding acquisition:** Yu Wang.

**Investigation:** Yu Wang.

**Writing – original draft:** Yu Wang.

**Writing – review & editing:** Yu Wang.
